# Body appreciation is associated with optimism/pessimism in patients with chronic kidney disease: Results from a cross-sectional study and validation of the Arabic version of the Optimism–Pessimism Short Scale–2

**DOI:** 10.1371/journal.pone.0306262

**Published:** 2024-09-06

**Authors:** Maroun Hajj-Moussa, Nay El Hachem, Ziad El Sebaaly, Perla Moubarak, Reine-Marie Kahwagi, Diana Malaeb, Rabih Hallit, Sami El Khatib, Souheil Hallit, Sahar Obeid, Feten Fekih-Romdhane

**Affiliations:** 1 School of Medicine and Medical Sciences, Holy Spirit University of Kaslik, Jounieh, Lebanon; 2 Department of Nephrology, Bhannes Medical Center, Bhannes, Lebanon; 3 College of Pharmacy, Gulf Medical University, Ajman, United Arab Emirates; 4 Department of Infectious Disease, Bellevue Medical Center, Mansourieh, Lebanon; 5 Department of Infectious Disease, Notre Dame des Secours, University Hospital Center, Byblos, Lebanon; 6 Department of Biomedical Sciences, School of Arts and Sciences, Lebanese International University, Bekaa, Lebanon; 7 Center for Applied Mathematics and Bioinformatics (CAMB), Gulf University for Science and Technology (GUST), Hawally, Kuwait; 8 Department of Psychology, College of Humanities, Effat University, Jeddah, Saudi Arabia; 9 Applied Science Research Center, Applied Science Private University, Amman, Jordan; 10 Social and Education Sciences Department, School of Arts and Sciences, Lebanese American University, Jbeil, Lebanon; 11 The Tunisian Center of Early Intervention in Psychosis, Department of psychiatry “Ibn Omrane”, Razi Hospital, Manouba, Tunisia; 12 Tunis El Manar University, Faculty of Medicine of Tunis, Tunis, Tunisia; National University of Medical Sciences, PAKISTAN

## Abstract

**Background:**

Chronic Kidney Disease (CKD) patients encounter many obstacles that affect their physical and psychological well-being. The primary objective of the present study was to investigate potential correlates of optimism/pessimism in a sample of patients with CKD, including socio-demographics, body appreciation and disordered eating symptoms. As a secondary objective, we proposed to examine the psychometric properties of an Arabic translation of the Optimism–Pessimism Short Scale–2 (SOP2) before its use in our sample.

**Methods:**

A cross-sectional study was carried out between April and May 2023, enrolling 108 participants from three hospitals in Lebanon providing insights into their sociodemographic characteristics, physical activity levels, body appreciation, optimism, pessimism, and eating attitudes.

**Results:**

Results indicated that the Arabic-language adaptation of the SOP2 has good reliability coefficients. The two facets of the scale displayed a strong correlation to each other, and highly similar correlations with external study variables (i.e., household crowding index, physical activity, body appreciation, disordered eating), suggesting that the SOP2 can be interpreted as a unidimensional measure for the psychological dispositional optimism as proposed by the developers. Furthermore, findings revealed a strong positive correlation between body appreciation and optimism, suggesting that CKD patients who appreciate their bodies tend to exhibit a more optimistic outlook on life. Conversely, higher body appreciation is associated with lower pessimism, indicating that a positive body image may mitigate feelings of hopelessness and despair in CKD patients.

**Conclusion:**

This study contributes to the literature in two ways. First, it offers the Arabic SOP2 as an brief tool to administer, and psychometrically sound instrument that can be useful for clinical and research purposes. Second, it unveils a clear correlation between higher body appreciation and a more optimistic, less pessimistic mindset in patients with CKD.

## Introduction

Individuals with Chronic Kidney Disease (CKD) face significant challenges in their daily lives, including a profound impact on their mental health [1]. For example, dealing with a chronic illness can trigger emotions of unease, despair, and ambiguity [2]. In addition, the relentless need for medical appointments, dietary restrictions, and medication regimens may create a constant state of stress and emotional strain [3]. Furthermore, the looming prospect of dialysis or transplantation may exacerbate feelings of vulnerability and distress [4]. In turn, the impact of CKD on social and family life can lead to isolation and reduced quality of life [2]. Having a general predisposition toward optimism can be an important source of strength that helps patients cope with the disease, face disease-related challenges, and prevent negative outcomes [5].

### Optimism in patients with CKD

Dispositional optimism, the tendency to believe that future expectations or outcomes will be favorable [6] is a positive psychological asset that may be an effective coping strategy against environmental stressors, even if illusory or unrealistic [7]. Thus, optimism, a positive mental attitude characterized by hopefulness and confidence in the face of challenges, has long been recognized as a crucial factor in shaping one’s overall well-being and health outcomes [8]. It plays a significant role in influencing how individuals cope with adversity and how they perceive and respond to life’s ups and downs [9]. Moreover, over the years, researchers have delved into the profound impact of optimism on various health conditions, including chronic diseases [10]. On the other hand, pessimism, the tendency to have a negative outlook and expect unfavorable outcomes in various situations, can profoundly impact a person’s mental and physical well-being [11]. There is evidence that optimism motivates the individual to take proactive measures to protect his/her health, while pessimism is associated with behaviors that are adverse to health [12]. On the other hand, studies such as those by Cohen et al. (1999) [13] and Segerstrom (2005) [14] have shown that when stressors are short-lived (i.e., less than a week) optimism appears to be protective against the effects of stress. However, this effect is reversed when the stressors are prolonged, as optimists are more immunologically vulnerable under such circumstances [13, 14].

While optimism has been extensively studied with CKD, the role of pessimism in this context is equally significant but less explored [15]. Chronic kidney disease is a widespread health condition affecting millions of people worldwide [16]. Understanding the influence of optimism and pessimism on its development, progression, and management is crucial for comprehensive patient care [17]. A study found that optimism was tightly linked to cardiovascular outcomes where people with optimistic thoughts had positive cardiovascular outcomes. In addition, mindfulness-based programs and positive interventions are proven techniques to boost psychological well-being [18]. Moreover, Yue et al demonstrated that optimism and health behaviors were broadly and robustly associated with a lower risk of mortality [19]. For example, patients on hemodialysis who have more optimistic thoughts may tend to take their medications on time and comply with their treatment along with the diet recommendation concerning their disease. Additionally, Lopez-Vargas et al. (2014) [20] studied a focus group and reported that the patients diagnosed with chronic kidney disease believed that a positive and optimistic perspective on life enabled them to better deal with their diseases and that hope for a better future led them to feel more encouraged to implement changes in their lifestyles. Adding to that, optimism may be related to favorable kidney disease outcomes because it is an effective coping strategy. Hence, it is considered a marker of resilience, and is linked to lower levels of distress, effective problem solving, personal success, and better perceived health [21]. People who are more optimistic tend to have better self-regulation (increasing personal efforts when circumstances are favorable and decreasing efforts when circumstances are unfavorable) and better diets (less inappropriate eating) [22].

### Measures of optimism

As dispositional optimism has consistently proven to be related to a wide range of personal and life outcomes, the construct has attracted research interest and measures have been developed for its assessment such as the Life Orientation Test-Revised (LOT-R; [23]). In 2013, Kemper et al. [24] designed and validated an ultrashort scale, the two-item Optimism–Pessimism Short Scale–2 (SOP2), that detects the key aspects of dispositional optimism as defined by Scheier and Carver [25]. The scale is composed of two items assessing generalized positive (optimism) and negative (pessimism) expectations about the future. The SOP2 has shown good psychometric qualities in a random and representative sample of German adults and can be regarded as a bipolar measure for dispositional optimism with a single factor structure [24]. Later, the SOP2 was adapted to the English language, and validated in a sample of UK adults [26]. To our knowledge, no Arabic version of any dispositional optimism measure, including the SOP2, exists to date.

### Correlates of optimism

Levels of optimism/pessimism seem to depend on multiple demographic factors. In particular, the intricate relationship between gender, socioeconomic status, physical activity, and optimism-pessimism bears profound psychological significance, warranting exploration within the medical context [27]. Furthermore, optimism, pessimism, and age intertwine in human psychology [28]. For example, youth often embraces optimism, driven by invincibility and potential [29]. With age, experiences may foster pessimism and caution [30]. However, individual differences exist, with some maintaining optimism and others developing a positive outlook in later years [31]. Understanding these dynamics can aid healthcare professionals in providing comprehensive emotional care across different life stages. A previous study on African-American patients found that the more optimism was associated with lower odds of CKD and lower odds of rapid kidney function deterioration [32]. Extensive research indicates that gender disparities exert influence over optimism levels, with the female gender generally manifesting a predisposition towards higher levels of optimism compared to males [33]. Furthermore, socioeconomic status emerges as a critical determinant, with individuals hailing from more privileged backgrounds exhibiting a propensity towards optimistic outlooks [34]. Notably, physical activity assumes a pivotal role in shaping one’s disposition, as regular engagement in exercise has been associated with heightened optimism, attributable to its positive impact on mood and overall well-being. In contrast, a sedentary lifestyle may render individuals more susceptible to pessimism [35].

Other possible correlates of optimism/pessimism in the particular context of CKD could be body appreciation [36] and disordered eating [37]. The nuanced association between body appreciation and eating behaviors underscores significant psychological implications and its interrelation with optimism and pessimism, needing further investigations in the medical context [38]. Body dissatisfaction may present itself among individuals diagnosed with chronic kidney disease, as they struggle with the physical alterations that accompany their medical condition [39]. Individuals with CKD may experience fluid retention, weight fluctuations, and changes in body composition, which can result in negative body image and reduced self-esteem [40]. These individuals may experience a sense of disconnect between their perceived self-image and their pre-CKD body, which can contribute to emotional distress and impact their overall quality of life [41].

Enhanced body appreciation is linked to the adoption of healthier eating behaviors, prioritizing self-nourishment and care [42]. This positive body image further correlates with a greater sense of optimism, characterized by a confident and hopeful outlook on life [43]. Conversely, individuals experiencing negative body image and dissatisfaction may be prone to engaging in maladaptive eating patterns, such as binge eating or restrictive diets, potentially exacerbating pessimistic tendencies [44–46].

### The present study

Studies correlating the three variables together are scarce in the literature, specifically among CKD patients. In the literature, only one cross-sectional study carried out among female students has focused on dispositional optimism and binge eating, and has found a negative association [47]. Other bivariate data available between optimism and eating disorders indicated contrasted results [48]. In addition, these studies have been carried out among specific populations such as athletes or undergraduate students, with no target of specific populations.

By examining the interplay between optimism, pessimism, and multiple independent variables in the context of CKD, this study is of concern because it will provide a comprehensive understanding of the psychosocial aspects of the disease. The findings from this research can inform the development of targeted interventions and support systems tailored to CKD patients’ unique needs. Identifying modifiable factors related to optimism and pessimism can aid healthcare professionals in providing more personalized patient care, fostering emotional resilience, and improving overall health outcomes. Uncovering potential associations between optimism, pessimism, and the independent variables may identify individuals at higher risk of experiencing psychological distress or poor disease outcomes. In addition to what was written using an optimistic approach with patients having chronic kidney disease might help them comply with their treatment along with a better prognosis. Thus, trying to implement an optimistic approach in patient care might increase rates of survival. Moreover, working early on patients to implement optimism and limit pessimism might reduce their daily stress and empower them with their daily disability, they might increase their coping approach with their limitations and might reduce their risk of developing serious complications of the disease.

The prevailing negative events in Lebanon have exacerbated the level of pessimism among its population, and one particularly distressing aspect is the dire situation facing chronic kidney disease patients in their access to essential medical care [49]. Amidst economic turmoil, the healthcare system in Lebanon has crumbled, leading to severe shortages of medications, medical supplies, and qualified healthcare professionals [50]. For CKD patients, this has translated into an alarming struggle to secure life-sustaining dialysis treatments, regular check-ups, and access to necessary medications. The uncertainty surrounding their healthcare, coupled with financial hardships, has created an atmosphere of despair, intensifying the already overwhelming pessimism gripping the nation.

This information can help prioritize support and resources for vulnerable patients and guide proactive interventions. As no previous study has comprehensively explored the relationship between optimism, pessimism, and body appreciation in CKD patients, this research contributes to the development of new knowledge in the field. It opens the door for future investigations and offers a pioneering perspective in understanding the psychosocial dimensions of CKD. The primary objective of the present study was to investigate potential correlates of optimism/pessimism in a sample of patients with CKD, including socio-demographics, body appreciation and disordered eating symptoms. As a secondary objective, we proposed to examine the psychometric properties of an Arabic translation of the SOP2 [51] before its use in our sample.

## Methods

### Study design and procedure

This cross-sectional study was carried out between April and May 2023, enrolling a total of 108 participants from three hospitals in Lebanon. The snowball sampling technique was employed to select participants for our research. To facilitate data collection, we developed a digital version of the questionnaire using Google Forms software, opting for an online approach for ease and accessibility. The snowball sampling technique was used because of the immunocompromised state of the patients having CKD, therefore, the link was sent to the patients entering the dialysis center and to nephrologists in their clinics. Before participation, all participants were informed about the study’s objectives and provided with clear instructions for completing the questionnaire through an online platform. We rigorously examined Internet Protocol (IP) addresses to ensure data integrity to prevent duplicate survey submissions and maintain data validity. Notably, no incentives or rewards were offered to participants, ensuring that responses were entirely voluntary. Our study included all patient’s male or female 18 and older with chronic kidney disease, patients presenting to the hemodialysis center of these hospitals, and all patients presented to the clinics of the center with abnormal creatinine levels. Hence, we included patients self-reporting as they have a chronic kidney disease, and excluded those who reported not presenting any kidney disease.

### Ethics approval and consent to participate

Ethics approval for this study was obtained from the Research Committee from the School of Pharmacy at the Lebanese International University (2023RC-017-LIUSOP). Written informed consent was obtained from all subjects; the online submission of the soft copy was considered equivalent to receiving written informed consent. All methods were performed in accordance with the relevant guidelines and regulations.

### Minimal sample size calculation

Assuming an R^2^ deviation from zero of .15, an alpha error of 5%, a power of 80%, and 9 predictors to enter in the multivariable analysis, the G-power software calculated a minimum sample size of 98 patients needed to have enough statistical power.

### Questionnaires

The questionnaires included various sections to gather relevant data. Firstly, participants were provided with a clear explanation of the study’s topic and objectives. They were assured of the anonymity of their responses and informed about the importance of offering their informed consent before participation. The subsequent section focused on collecting sociodemographic information from participants, encompassing factors such as age, gender, marital status, level of education, and self-reported current weight and height. Using this data, the Body Mass Index (BMI) was calculated according to the World Health Organization (WHO) guidelines [52]. The household crowding index was computed to assess the socioeconomic status of participants’ households [53]. This index is determined by dividing the total number of individuals residing in the household by the total number of rooms in the dwelling, excluding kitchen and bathrooms. Additionally, participants’ physical activity levels were evaluated using the physical activity index, which takes into account the combined result of daily activity intensity, duration, and frequency [54]. This comprehensive measure provides valuable insights into participants’ overall physical activity levels.

*The Optimism–Pessimism Short Scale–2 (SOP2)* [51], measures the psychological disposition of optimism with two items rated on a seven-point Likert scale from not at all optimistic (1) to very optimistic (7) for item 1 and from not at all pessimistic (1) to very pessimistic (7) for item 2. To obtain an optimism scale score, the negatively worded item is recorded, and the unweighted mean score of the two items is computed (e.g., The next question deals with optimism. Optimists are people who look to the future with confidence and who mostly expect good things to happen. How would you describe yourself? How optimistic are you in general?). The process of forward and backward translation was applied to the SOP2. First, the English version was translated into Arabic by a Lebanese translator who had no prior involvement with the study. Subsequently, a Lebanese psychologist, proficient in English, translated the Arabic version back into English. Both the initial English version and the translated one were carefully compared to identify and rectify any discrepancies. This review was carried out by a committee comprised of the research team and the two translators. Before commencing the official data collection, a pilot study involving 20 individuals was conducted to ensure the clarity and understanding of the questions. No modifications were deemed necessary as a result of this pilot study.

*The Eating Attitudes Test (EAT-7)*, previously validated in Arabic [55], was employed to assess the presence of disordered eating based on attitudes, emotions, and behaviors related to eating. The questionnaire consists of seven questions, each offering six response options, ranging from infrequent occurrences/almost never/never (scored as 0) to constant experiences (scored as 3). The total score is obtained by summing all responses ranging from 0 to 21. The higher the score, the more the inappropriate eating attitudes (Cronbach’s α = .76).

*Body Appreciation Scale (BAS-2)*, validated in Lebanon [56], this instrument comprises 10 items and measures an individual’s level of acceptance, respect, and care toward their body and their ability to shield themselves from unrealistic beauty standards [57]. Participants rate each item on a 5-point scale, ranging from "never" to "always." Elevated scores on this scale indicate a greater degree of body appreciation (Cronbach’s α = .96).

### Statistical analysis

The SPSS software v.26 was used for the statistical analysis. To assess the psychometric properties of the SOP2 scale, an exploratory factor analysis was executed; after confirming sample adequacy with the Kaiser–Meyer–Olkin (KMO) index and Bartlett’s test of sphericity, factors were extracted using a principal component analysis method. We retained factors with an Eigenvalue higher than one; we confirmed their adequacy with a Scree plot. Items with factor loading > 0.4 were considered as loading on a factor. We also checked the reliability using Cronbach’s alpha values for all scales. The optimism and pessimism scores were considered normally distributed as their skewness and kurtosis values varied between ± 1. The Student t-test was used to compare two means. Pearson test was used to correlate two continuous variables. Linear regressions were conducted afterward, taking the optimism and pessimism scores as dependent variables; factors with a *p* < .25 in the bivariate analysis were entered as independent variables in the regression models. *P* < .05 was deemed statistically significant.

## Results

### Sociodemographic characteristics

One hundred eight patients enrolled in the study, with a mean age of 63.76 years and 73.1% males. Other characteristics of the patients can be found in **Table 1.**

**Table 1:** Sociodemographic and other characteristics of the patients (n = 108).

	n (%)
**Gender**	
Males	79 (73.1%)
Females	29 (26.9%)
**Marital status**	
Single	30 (27.8%)
Married	78 (72.2%)
**Education**	
Primary	25 (23.1%)
Complementary	20 (18.5%)
Secondary	36 (33.3%)
University	27 (25.0%)
	**Mean ± SD**
Age (years)	63.76 ± 12.68
Body Mass Index (kg/m^2^)	24.56 ± 4.76
Household crowding index	1.03 ± .58
Physical activity	20.80 ± 21.69
Body appreciation	39.02 ± 9.41
Optimism	4.26 ± 1.68
Pessimism	3.83 ± 1.73
Disordered eating	3.39 ± 4.00

### Factor analyses

In the total sample, the items converged over a solution of one factor (variance explained = 84.44%; KMO = .500; Bartlett’s test of sphericity p < 0.001). The same was found in males and females respectively **Table 2.** The Cronbach’s alpha values were .79 in the total sample, .79 in males and .79 in females.

**Table 2:** Principal analysis component of the optimism-pessimism scale.

	Total sample	Males	Females
Optimism	.92	.92	.92
Pessimism	-.92	-.92	-.92
Variance explained	84.44%	85.10%	84.08%

### Bivariate analyses

The results of the bivariate analysis are summarized in **Tables 3 and 4**. Optimism was not significantly associated with gender (t(106) = .71, p = .477), marital status (t(106) = -.10, p = .921), or education F(3, 104) = 1.64, p = .186). Pessimism was not significantly associated with gender (t(106) = .40, p = .692), marital status (t(106) = .25, p = .805), or education F(3, 104) = .87, p = .461). Finally, higher body appreciation was significantly associated with higher optimism and lower pessimism.

**Table 3:** Factors associated with optimism and pessimism.

	Optimism	*p*	Pessimism	*p*
**Gender**		.477		.692
Males	4.33 ± 1.65		3.87 ± 1.81	
Females	4.07 ± 1.75		3.72 ± 1.51	
**Marital status**		.921		.805
Single	4.23 ± 1.63		3.90 ± 1.69	
Married	4.27 ± 1.70		3.81 ± 1.75	
**Education**		.186		.461
Primary	3.68 ± 1.89		4.24 ± 1.76	
Complementary	4.20 ± 1.44		3.67 ± 1.47	
Secondary	4.39 ± 1.36		3.56 ± 1.91	
University	4.67 ± 1.94		3.83 ± 1.73	

**Table 4:** Correlation matrix of continuous variables.

	1	2	3	4	5	6	7
1. Optimism	1						
2. Pessimism	-.69***	1					
3. Age	-.10	.04	1				
4. Household crowding index	-.13	.14	-.31**	1			
5. Physical activity	.18	-.16	-.40***	.22*	1		
6. Body appreciation	.31**	-.34***	.06	-.04	.06	1	
7. Disordered eating	.16	-.10	-.02	.07	-.06	.06	1

*p < .05

**p < .01

***p < .001

### Multivariate analyses

The first model explained 19.3% of the variability of the optimism score (R^2^ = .193). Body appreciation predicted optimism, F(7, 100) = 3.41, p = .002, 95% CI [.20, .85].

The second model explained 18.9% of the variability of the pessimism score (R^2^ = .189). Body appreciation was associated with less pessimism, F(7, 100) = 3.33, p = .003, 95% CI [-.09; -.02] **Table 5**.

**Table 5:** Multivariable analyses.

	Unstandardized Beta		Standardized Beta	*p*	95% CI
**Model 1: Optimism as the dependent variable (R**^**2**^ **= .193)**					
Complementary education level vs primary*		.28	.06	.560	-.66; 1.21
Secondary education level vs primary*		.56	.16	.176	-.25; 1.37
University education level vs primary*		.71	.18	.112	-.17; 1.59
Household crowding index		-.39	-.14	.156	-.94; .15
Physical activity		.01	.17	.076	-.001; .03
Body Mass Index		-.05	-.13	.171	-.11; .02
Body appreciation		.05	.30	**.002**	.02; .09
**Model 2: Pessimism as the dependent variable (R**^**2**^ **= .189)**					
Complementary education level vs primary*	-.02		-.004	.972	-.98; .95
Secondary education level vs primary*	-.44		-.12	.296	-1.28; .39
University education level vs primary*	-.47		-.12	.310	-1.38; .44
Household crowding index	.48		.16	.091	-.08; 1.04
Physical activity	-.01		-.18	.057	-.30; .001
Body appreciation	-.06		-.31	**.001**	-.09; -.02
Disordered eating	-.51		-.12	.206	-1.32; .29

*Reference group; numbers in bold indicate significant *p* values.

## Discussion

The present study investigates, for the first time, the interactions between body appreciation, disordered eating symptoms and levels of optimism/pessimism in the specific population of patients diagnosed with CKD. The results showed that increased body appreciation was associated with higher optimism and lower pessimism in CKD patients in Lebanon. Moreover, the SOP2 translation and validation suggested that it can be applicable and suitable for use among Arabic-speaking people.

### The psychometric properties of the Arabic SOP2

The results of the analysis for the Arabic version of the Optimism Pessimism Short Scale 2 (SOP2) support adequate psychometric properties of the instrument in the present sample.

Consistent with the English- [51] and German-language [58] validation studies of the scale, results indicated that the Arabic-language adaptation of the SOP2 has good reliability coefficients. The two facets of the scale displayed strong correlation to each other, and highly similar correlations with external study variables (i.e., household crowding index, physical activity, body appreciation, disordered eating), suggesting that the SOP2 can be interpreted as a unidimensional measure for the psychological dispositional optimism as proposed by the developers [58]. In addition, findings demonstrated that the Arabic SOP2 correlated with positive aspects of body image, thus providing additional evidence that dispositional optimism is closely connected to numerous indicators of psychological health (e.g., [59]). All these results indicate that the Arabic SOP2 is a brief tool to administer, and psychometrically sound instrument that can be useful for clinical and research purposes.

### Body appreciation and optimism

The results of our study demonstrated that body appreciation was associated with higher optimism. These results are in confirmation of a previous study where optimism and positive body appreciation were associated with each other in a sample of women [36]. Moreover, body appreciation is closely linked to higher self-esteem [60]. When people learn to appreciate their bodies, they typically develop a more positive self-image [61], leading to a boost in self-esteem that lays the foundation for greater optimism in life [62]. In addition, people with higher self-esteem are more likely to face challenges with resilience, maintain a hopeful outlook on the future, and believe in their own abilities [63].

As well, numerous studies have shown a strong connection between body appreciation and improved mental health [64]. When people feel good about their bodies, they are less likely to experience symptoms of anxiety and depression [65]. Furthermore, this improved mental well-being creates a more optimistic outlook on life, as individuals are better equipped to cope with stress and life’s challenges [66]. Additionally, building body appreciation can lead to more positive social interactions. Hence, when individuals are confident and secure in their bodies, they tend to radiate positivity, which can attract like-minded individuals [67]. Likewise, strong social connections are a key factor in fostering optimism, as they provide a support system during challenging times [68]. In addition to what was stated, the Jackson heart study demonstrated that optimistic thoughts are associated with lower odds of developing kidney disease and lower odds of rapid kidney function decline [32]. Chan et al. demonstrated that persons having higher levels of optimism exhibited less cortisol secretion in the awakening period when the effect of pessimism and mood were controlled [69]. This might explain why increased levels of stress and pessimism can alter kidney function and make their prognosis worse.

### Body appreciation and pessimism

This study showed that higher body appreciation was associated with lower pessimism among CKD patients. These results were unique because no studies were done on the relation between pessimism and body appreciation. Low body appreciation can contribute to psychological distress in CKD patients. When individuals have a negative view of their bodies, it may lead to feelings of sadness, anxiety, and a sense of hopelessness [70]. This psychological distress can contribute to an overall pessimistic outlook on life [71]. In addition, CKD patients with low body appreciation may experience a reduced quality of life [72] by limiting social interactions, hindering participation in activities, and eroding self-confidence [73]. These limitations can exacerbate feelings of pessimism and hinder patients’ ability to cope with the challenges of CKD effectively.

Moreover, CKD patients’ attitude toward their bodies can significantly impact their adherence to treatment [74]. Patients who possess a positive body image are more likely to remain committed to their treatment plans, which typically involve dietary restrictions, medication, and dialysis [75]. Conversely, individuals with low body appreciation may struggle to comply with their prescribed regimen, resulting in potentially severe health consequences and a pessimistic outlook for the future [75].

### Clinical implications

The study of the connection between body appreciation and optimism/pessimism among Lebanese patients with chronic kidney disease has unveiled significant clinical implications. Healthcare professionals need to offer comprehensive psychosocial support that includes interventions targeting body image perception. Consistent mental health screening and a multidisciplinary approach are essential in identifying patients vulnerable to body dissatisfaction and pessimism. Providing patients with adaptive coping strategies, tailored to Lebanon’s unique sociocultural and geopolitical context, is crucial. Additionally, improving the healthcare infrastructure is key in mitigating the stressors that contribute to pessimism. An evidence-based clinical landscape is vital, relying on rigorous research and data aggregation to provide superior care for CKD patients in the challenging Lebanese healthcare environment.

### Limitations

This study has several notable limitations that must be acknowledged. Firstly, due to its cross-sectional design, we cannot establish a causal relationship between the variables examined. Additionally, the potential for information bias exists, as participants may not have fully comprehended the questions in the self-administered online questionnaire. Furthermore, the questionnaire’s length may have impacted participants’ responses, as they may not have remained fully engaged in a survey lasting more than 10 minutes. Moreover, the presence of selection bias cannot be ruled out, most individuals in our sample were males who were married. Consequently, the findings of this study may not effectively capture variations related to gender or socioeconomic status among the analyzed variables. Lastly, it’s worth mentioning that confounding bias may be present since not all factors associated with optimism and pessimism were considered in this study.

## Conclusion

The findings of this study have shed light on the correlation between body appreciation and outlook in those with chronic kidney disease. Specifically, it has been shown that a greater degree of body appreciation was linked to a more optimistic and less pessimistic mindset. This highlights the significance of exploring this relationship in more detail, particularly among specific populations, including students, employees, and clinical patients, as well as across various age groups. Further research in this area could provide valuable insights into how individuals’ perceptions of their own bodies impact their overall mental health and well-being.

## Supporting information

S1 Checklist Human participants research checklist. (DOCX)

S2 Checklist STROBE statement -checklist of items that should be included in reports of observational studies. (DOCX)
