# Supplementary material for: Body appreciation is associated with optimism/pessimism in patients with chronic kidney disease: Results from a cross-sectional study and validation of the Arabic version of the Optimism–Pessimism Short Scale–2
Source: PLoS One. 2024 Sep 6;19(9):e0306262. doi: 10.1371/journal.pone.0306262 (PMC11379166; doi:10.1371/journal.pone.0306262)
Supplement: S2 Checklist — (DOCX) [file pone.0306262.s002.docx]

**STROBE**

STROBE Statement -checklist of items that should be included in reports of observational studies

| **Section/topic** | **Item No.** | **Recommendation** | **Page No.** |
| --- | --- | --- | --- |
| **Title and abstract** | 1 | (a) Indicate the study’s design with a commonly used term in the title or the abstract | 3 |
|  |  | (b) Provide in the abstract an informative and balanced summary of what was done and what was found | 3 |
| **Introduction** | | | |
| Background/ rationale | 2 | Explain the scientific background and rationale for the investigation being reported | 4-7 |
| Objectives | 3 | State specific objectives, including any prespecified hypotheses | 7 |
| **Methods** | | | |
| Study design | 4 | Present key elements of study design early in the paper | 7 |
| Setting | 5 | Describe the setting, locations, and relevant dates, including periods of recruitment, exposure, follow-up, and data collection | 7-8 |
| Participants | 6 | (a) **Cohort study** -Give the eligibility criteria, and the sources and methods of selection of participants. Describe methods of follow-up  **Case-control study** -Give the eligibility criteria, and the sources and methods of case ascertainment and control selection. Give the rationale for the choice of cases and controls  **Cross-sectional study** -Give the eligibility criteria, and the sources and methods of selection of participants | 7-8 |
|  |  | (b) **Cohort study** -For matched studies, give matching criteria and number of exposed and unexposed  **Case-control study** -For matched studies, give matching criteria and the number of controls per case |  |
| Variables | 7 | Clearly define all outcomes, exposures, predictors, potential confounders, and effect modifiers. Give diagnostic criteria, if applicable | 8-9 |
| Data sources/ measurement | 8* | For each variable of interest, give sources of data and details of methods of assessment (measurement). Describe comparability of assessment methods if there is more than one group | 8-9 |
| **Section/topic** | **Item No.** | **Recommendation** | **Page No.** |
| Bias | 9 | Describe any efforts to address potential sources of bias | 16 |
| Study size | 10 | Explain how the study size was arrived at | 8 |
| Quantitative variables | 11 | Explain how quantitative variables were handled in the analyses. If applicable, describe which groupings were chosen and why | 9-10 |
| Statistical methods | 12 | (a) Describe all statistical methods, including those used to control for confounding | 9-10 |
|  |  | (b) Describe any methods used to examine subgroups and interactions | N/A |
|  |  | (c) Explain how missing data were addressed | 9-10 |
|  |  | (d) **Cohort study** -If applicable, explain how loss to follow-up was addressed  **Case-control study** -If applicable, explain how matching of cases and controls was addressed  **Cross-sectional study** -If applicable, describe analytical methods taking account of sampling strategy | N/A |
|  |  | (e) Describe any sensitivity analyses | N/A |
| **Results** | | | |
| Participants | 13* | (a) Report numbers of individuals at each stage of study—eg numbers potentially eligible, examined for eligibility, confirmed eligible, included in the study, completing follow-up, and analysed | 10 |
|  |  | (b) Give reasons for non-participation at each stage | N/A |
|  |  | (c) Consider use of a flow diagram | N/A |
| Descriptive data | 14* | (a) Give characteristics of study participants (eg demographic, clinical, social) and information on exposures and potential confounders | 10 |
|  |  | (b) Indicate number of participants with missing data for each variable of interest | 9-10 |
|  |  | (c) **Cohort study** -Summarise follow-up time (eg, average and total amount) | N/A |
| Outcome data | 15* | **Cohort study** -Report numbers of outcome events or summary measures over time  **Case-control study** -Report numbers in each exposure category, or summary measures of exposure  **Cross-sectional study** -Report numbers of outcome events or summary measures | 11-13 |
| **Section/topic** | **Item No.** | **Recommendation** | **Page No.** |
| Main results | 16 | (a) Give unadjusted estimates and, if applicable, confounder-adjusted estimates and their precision (eg, 95% confidence interval). Make clear which confounders were adjusted for and why they were included | 10-13 |
|  |  | (b) Report category boundaries when continuous variables were categorized | N/A |
|  |  | (c) If relevant, consider translating estimates of relative risk into absolute risk for a meaningful time period | N/A |
| Other analyses | 17 | Report other analyses done -eg analyses of subgroups and interactions, and sensitivity analyses | N/A |
| **Discussion** | | | |
| Key results | 18 | Summarise key results with reference to study objectives | 13 |
| Limitations | 19 | Discuss limitations of the study, taking into account sources of potential bias or imprecision. Discuss both direction and magnitude of any potential bias | 16 |
| Interpretation | 20 | Give a cautious overall interpretation of results considering objectives, limitations, multiplicity of analyses, results from similar studies, and other relevant evidence | 13-15 |
| Generalisability | 21 | Discuss the generalisability (external validity) of the study results | 16 |
| **Other information** | | | |
| Funding | 22 | Give the source of funding and the role of the funders for the present study and, if applicable, for the original study on which the present article is based | 17 |

* Give information separately for cases and controls in case-control studies and, if applicable, for exposed and unexposed groups in cohort and cross-sectional studies.
